# Idiopathic benign retroperitoneal cyst: a case report

**DOI:** 10.1186/1752-1947-2-43

**Published:** 2008-02-08

**Authors:** Ahmed Alzaraa, Husam Mousa, Paul Dickens, Jonathan Allen, Abduljalil Benhamida

**Affiliations:** 1 Department of General Surgery, Tameside General Hospital, Manchester, UK; 2 Department of Histopathology, Tameside General Hospital, Manchester, UK

## Abstract

**Introduction:**

Retroperitoneal cysts are uncommon, with an estimated incidence of 1/5750 to 1/250,000.

**Case presentation:**

A male patient was admitted with an abdominal pain, jaundice and fever. Clinical examination and investigations confirmed an idiopathic benign retroperitoneal cyst. He underwent surgery and was discharged after making good recovery.

**Conclusion:**

Retroperitoneal cysts are very rare, and most of the time they are discovered incidentally. Patients may be asymptomatic or present with abdominal pain, referred pain to the legs or weight loss. Imaging may help diagnose these lesions, but surgery is the keystone in confirming the diagnosis. This case is very rare and very educational as it highlights an unusual presentation of a benign retroperitoneal cyst. In our patient, the course of the disease was unique as the patient presented with jaundice.

## Introduction

Retroperitoneal cysts (RPCs) are uncommon with an estimated incidence of 1/5750 to 1/250,000 [1]. Approximately one third of patients with retroperitoneal cysts are asymptomatic and the cyst is found incidentally. The cyst can grow to a considerable size before becoming symptomatic. CT scan might help in confirming the diagnosis, and surgery remains the best treatment option.

## Case presentation

A 51 year-old man was admitted on the surgical ward in November 2003 for a right-sided abdominal pain which had been present for three days. Clinically, he was jaundiced with a high temperature. Blood tests showed white cell count (20.8 × 10^3^/mm^3^), bilirubin (103 mg/dl) and ESR (75 mm/h). All other blood tests were normal. Ultrasound and CT scans of the abdomen revealed fluid collection/mass measuring 14 cm in the right hepato-renal space. The mass was separated from the liver, pancreas and the right kidney, but there was lack of definition of the right suprarenal gland. Because the patient was jaundiced, we thought that draining the cyst would relieve the pressure on the biliary system and resolve jaundice. About 150 ml brown-coloured fluid was aspirated from the mass under CT guidance and a sample was sent for histology. Cytological examination of the sample reported presence of amorphous material with occasional histiocytic cells. There was no evidence of malignancy. The patient was discharged in December 2003 after making good recovery.

**Figure 1:**
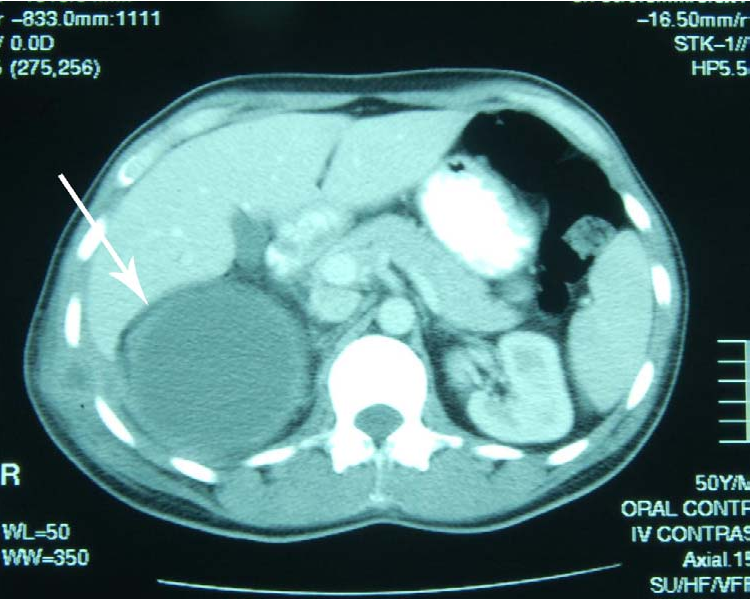
Retroperitoneal cyst (arrow) shown on abdominal CT.

In January 2004, the patient was readmitted for an abdominal pain and pyrexia. A repeat CT scan confirmed recollection of fluids at the same site (Figure 1). He underwent a laparotomy for excision of the mass and right adrenalectomy in February 2004. The specimen was sent for histopathology.

Macroscopical examination of the specimen reported an open cyst measuring 190 mm × 110 mm × 0.5 mm. The outer surface was pale-dark brown with an irregular defect measuring 1.8 cm × 1.5 cm in one area. Another area showed an adrenal tissue measuring 3.0 cm × 1.0 cm × 0.5 cm with yellow areas on the surface of the cyst wall. The inner area of the cyst wall was wrinkled with an exudate-like substance coating it in places.

**Figure 2:**
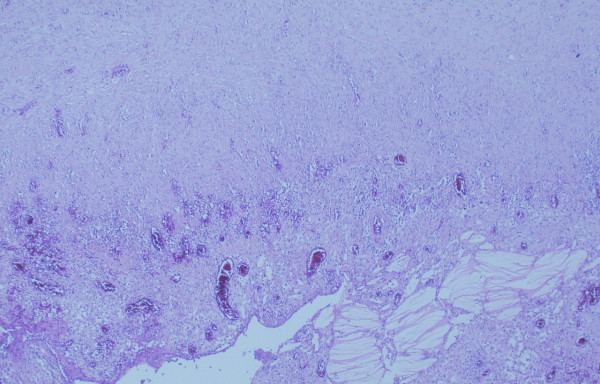
Mesenteric cyst wall showing fibrous wall granulation tissue lining with cholesterol crystal deposition.

**Figure 3:**
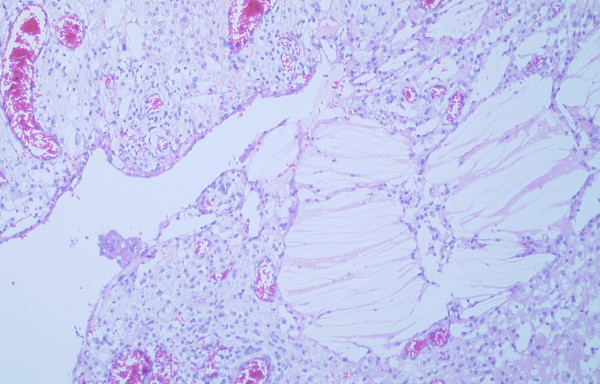
Mesenteric cyst wall with granulation tissue and cholesterol crystals deposition (H&E ×100).

Microscopically, the sections showed that the normal adrenal gland was adherent by fibrous tissues to the external wall of the cyst, but the cyst was not arising from the adrenal. The cyst wall consisted of a thick layer of fibrous tissues which showed focal calcifications and areas of acute and chronic inflammation. There was no epithelial lining present, but clusters of cholesterol crystals were adherent to the internal cyst wall. Granulation tissue also formed part of the lining of the cyst (Figures 2 &3). There was no atypia or malignancy. The overall appearances were those of an idiopathic benign retroperitoneal cyst.

## Discussion

Based on embryologic origin and histological differentiation, RPCs are classified into (a): Urogenital; (b): Mesocolic; (c): Cysts arising in cell inclusions; (d): Traumatic; (e): Parasitic and (f): Lymphatic [1,2]. Only those cysts that lie in the retroperitoneum without connection with any adult anatomical structure, except by areolar tissue, are included in this group of cysts [3]. The majority of urogenital cysts occur near the kidney, behind the colon, and near the head or tail of the pancreas. They arise from the vestiges of the embryonic urogenital apparatus and can be classified into pronephric, mesonephric, metanephric, and mullerian. When these cysts are exposed and seen in situ, they appear bluish thin-walled cysts and rather flabby with no visible vessels in their walls, and when removed, they are translucent and lose their bluish tinge. They have no pedicle and no connections apart from the areolar tissues with the surrounding structures. When opened, they have a smooth, glistening lining membrane; are single, not multilocular; and contain a clear serous fluid of low specific gravity in which there is occasionally cholesterin. Under the microscope, the wall is thin and consists of a cellular fibrous tissue usually lined by a low columnar epithelium, or cubical and rarely flattened [2]. The lymphatic cysts are subdivided into those formed in the lymphatics returning from the intestine and known as chylous cysts, and those arising in the lymphatic field behind the peritoneum and not connected with the intestine and are analogous in their origin to the single cystic lymphangioma seen in the head and neck [2]. They are unilocular or multilocular cysts containing clear or milky fluid and lined with a single layer of flattened endothelium [4]. Mesocolic are found only in the area between ascending and descending colon and below the transverse mesocolon, anterior to the spermatic or ovarian vessels, and are composed of a fibrous wall lined by a delicate flattened epithelium [2]. Cysts arising in cell inclusions such as dermoid cysts are found not infrequently in the retroperitoneum, have thick walls and usually filled with sebaceous material and hair [2]. Traumatic blood cysts may be due to haematoma resulting from an injury, ruptured abdominal aortic aneurysm, anticoagulant therapy, or blood dyscrasia, and they usually present as an emergency [4,2]. If the haematoma is not large enough, the development of a cyst is a well-recognised result [2]. Parasitic cysts such as hydatid cysts are not infrequent in the retroperitoneal space. They may reach this location by the blood stream, by transcaelomic implantation after the rupture of a cyst in the liver, or by penetrating the intestinal wall [2]. The complete differential diagnoses of retroperitoneal cysts in males and females is included (Table 1).

**Table 1:** Differential diagnoses of retroperitoneal cysts.

Female	Male
Lymphangioma	Lymphangioma
Cystic teratoma	Cystic teratoma
Cystic haematoma	Cystic haematoma
Cystic mesothelioma	Cystic mesothelioma
Bronchogenic cyst	Bronchogenic cyst
Epidermoid cyst	Epidermoid cyst
Tailgut cyst	Tailgut cyst
Mesenteric cyst	Mesenteric cyst
Pseudocyst (non-pancreatic)	Pseudocyst (non-pancreatic)
Pseudomyxoma peritonei	Pseudomyxoma peritonei
Urinoma	Urinoma
Lymphocele	Lymphocele
Endosalpingiosis	
Mullerian cyst	
Vulval cyst	
Parovaian cyst	
Vaginal cyst	
Paraurethral cyst	
Mucinous cystadenoma	

There are no pathognomonic signs or symptoms for RPCs, and in approximately one third of patients, the cyst is found incidentally [3,5]. Two thirds of patients present with an abdominal mass or chronic abdominal symptoms, most of them are omental in origin [3]. Other symptoms include back pain, referred pain to the lower limbs, oedema of the lower limbs, weight loss or fever [6,7]. The mass tend to be mobile in a transverse plane, or in all directions when the cyst is omental. More commonly, only a soft tissue mass with displacement of the bowel is seen [3].

CT is ideal for assessing RPCs because it provides discrete sectional images of the organs and retoperitoneal compartments, and in some case, familiarity with the most relevant radiologic features, in combination with clinical information, allows adequate lesion characterization [4]. Mullerian cyst, for example, manifest as a unilocular or multilocular thin-walled cyst containing clear fluid, and clinical history may help differentiate it from other retroperitoneal masses as it is more common in obese patients with menstrual irregularities [4]. A mature teratoma manifests as a complex mass containing a well-circumscribed fluid component, adipose tissue, and calcification. The presence of hypoattenuating fat within the cyst is considered highly suggestive of this cyst. The CT appearance of a retroperitoneal haematoma depends on the time elapsed between the traumatic event and imaging. Acute or subacute haematoma has a higher attenuation value than pure fluid due to clot formation. However, chronic haematoma has decreased attenuation because of the breakdown of blood products [4]. Cystic lymphangioma typically appears as a large, thin-walled, multiseptate cystic mass. Its attenuation values vary from that of fluid to that of fat. An elongated shape and a crossing from one retroperitoneal compartment to an adjacent one are characteristic of the mass, and calcification of the wall is rare [4].

Symptomatic cysts should be enucleated or excised, while preserving the surrounding vital structures. At times, the cyst can be marsupialised or drained if surgical enucleation is difficult or the cyst is infected [8]. However, draining the cyst usually result in a recurrence. In the analysis of the 162 patients who had mesenteric and RPCs, Kurtz R, et al [5] concluded that patients with RPCs were more likely to have incomplete excision of the cyst and therefore had a higher incidence of recurrence. They also required marsupialisation more often. Our patient should have had the cyst excised in the first place regardless of being jaundiced or not. Unfortunately, its pathogenesis was not known as the cyst did not have any epithelial lining.

## Conclusion

Cysts arising within the retroperitoneum outside the major organs within that compartment are very rare. Approximately one third of patients with retroperitoneal cysts are asymptomatic and the cyst is found incidentally. CT may help diagnose these lesions, but surgery remains the keystone in determining the diagnosis.

## Competing interests

The author(s) declare that they have no competing interests.

## Authors' contributions

A A: Reviewed literature and wrote the manuscript, H M: Contributed to the concept of the manuscript, P D: Evaluated histopathology, J A: Searched literature, A B: Operated on the patient. All authors have read and approved the manuscript.

Written informed consent was obtained from the patient for publication of this report and any accompanying images.

## References

[B1] Guile M Fagan M Simopolous A Ellerkman M Retroperitoneal Cyst of Mullerian Origin: A case report and review of the literature J of Pelvic Medicine and Surgery 2007 13 3 149 152 10.1097/SPV.0b013e3180622272

[B2] Handfield-Jones R Retroperitoneal Cysts: Their Pathology, Diagnosis and Treatment BJS 1942 119 134

[B3] Felix Edward L Wood Donald K Das Gupta Tapas K Tumours of the Retroperitoneun Cancer 1981 6 1 1 47 10.1016/s0147-0272(81)80011-6 6266775

[B4] Yang D Jung D Kim H Kang H Kim S Kim J Hwang H Retroperitoneal cystic masses: CT, Clinical and Pathological Findings and Literature Review RG 2004 25 5 1353 1365 10.1148/rg.245045017 15371613

[B5] Kurtz R Heimann T Beck R Holt J Mesenteric and Retroperitoneal Cysts Ann Surg 1986 203 1 109 112 10.1097/00000658-198601000-00017 3942415 PMC1251046

[B6] Haysaka Kazumasa Yamada Tomonori Saitoh Yasuhiro Yoshikawa Daihei CT Evaluation of Primary Benign Retropeitoneal Tumour Diagnostic Radiology 1994 12 3 115 120 7972894

[B7] Konishi Eiichi Nakashima Yasuaki Iwasaki Takeki Immunohistochemical analysis of Retroperitoneal Mullerian Cyst Human Pathology 2003 34 2 194 198 10.1053/hupa.2003.12 12612890

[B8] Ravo B Metwally N Pai B Ger R Developmental retroperitoneal Cysts of the Pelvis; A Review Dis Col & Rect 1987 30 7 559 564 10.1007/BF02554791 3595378

